# Observation of Plasmonics Talbot effect in graphene nanostructures

**DOI:** 10.1038/s41598-024-52595-2

**Published:** 2024-01-23

**Authors:** Ali Farmani, Anis Omidniaee

**Affiliations:** Department of Electronics Engineering, Lorestan University, Khoramabad, Iran

**Keywords:** Engineering, Materials science

## Abstract

We report on the theoretical models of the plasmoincs Talbot effect in graphene nanostructure. The Talbot effect for the plasmonics applications in the IR range is theoretically studied and the respective Talbot effect for the novel advanced plasmonics structures are numerically investigated for the first time. It is shown that the metamaterial structures with periodic grating configuration represents a complex three-dimensional lattice of beamlet-like graphene plasmonics devices. The calculated results agree well with the experimental ones. The results obtained can be used to create and optimize the structures considering diffraction limit for a wide range of application areas. Effective focusing of plasmonic waves with exact focal spots and a subwavelength full width at half maximum can be obtained by using periodic graphene grating.

## Introduction

The Talbot effect, also known as Talbot's law or Talbot self-imaging, is a phenomenon observed in optics. It was discovered by British scientist Henry Fox Talbot in the early nineteenth century. Talbot conducted experiments in which he placed a plane wavefront of light in front of a very thin screen pierced by regularly spaced parallel slits. He noticed that at certain distances from the screen, multiple images of the slits appeared. Talbot's investigations in 1836 led to the discovery of what is now called self-imaging or self-replication. He found that at specific distances from the slits, an exact replica of the original grating pattern would reappear. These distances were related to the wavelength of light and the period of the grating. This phenomenon has since been observed not only for slits, but also for various other types of periodic structures.

Initially, Talbot's findings didn't receive much attention. However, his work gained significance in the late twentieth century with the advent of laser technology. The Talbot effect has become invaluable in the field of optics and has applications in various areas, including the characterization of optical components, interferometry, and metrology. The Talbot effect is a result of Fresnel diffraction occurring in near fields. Upon the incidence of a light beam on an object with periodic geometry, any point on the surface of the object acts as a light source and creates an image at certain distances which is periodically repeated by increasing the distance from the image. The propagation of periodic wave can be under paraxial approximations and non-paraxial^1^.

**Figure 1:**
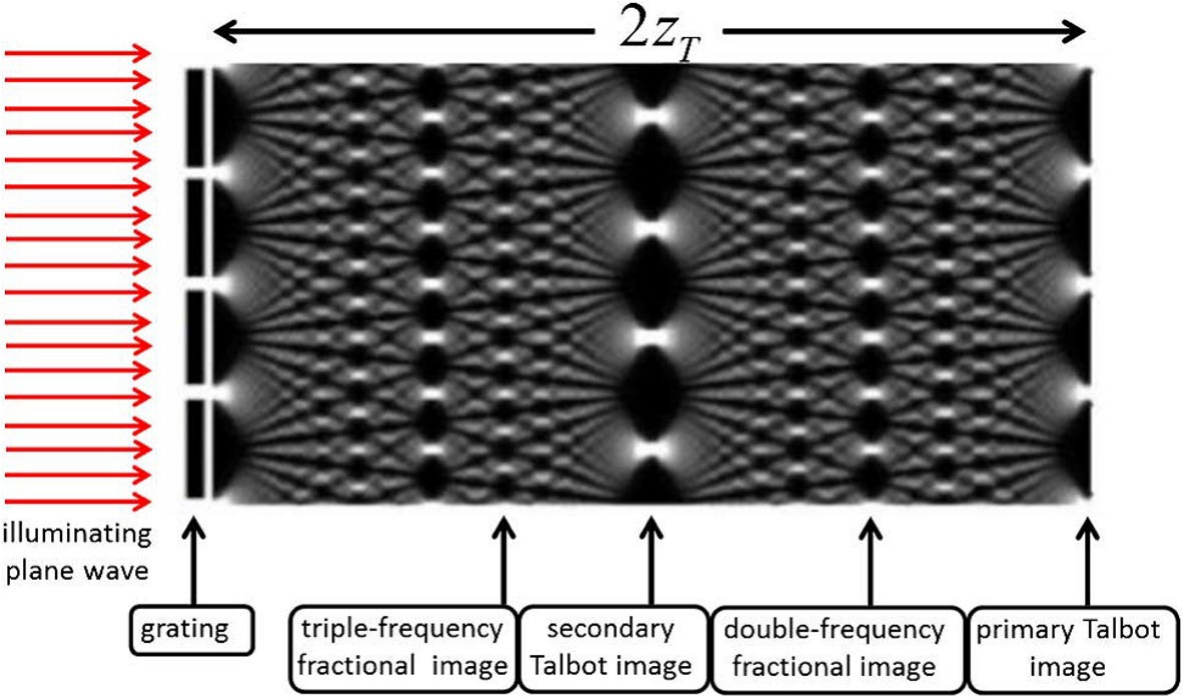
Observing the Talbot effect in a monochromatic wave^9^.

As can be seen in Fig. 1, the Talbot effect was first studied under paraxial approximation using the passage of a monochromatic light through a grating with regular slits where the image was repeated behind the grating at regular intervals. Shows the formation of the Talbot effect in a grating in one-dimensional space where an infinitely wide grating is illuminated by a monochromatic coherent wave. Non-paraxial approximations were recently assessed in plasmonic structures with longitude periodicity. By examining the Talbot effect for structures which are periodic in only one direction, Montgomery found that the required condition for the formation of Talbot image is periodicity in only one direction of the structure^2^. Nonparaxial propagation only includes a limited number of waves while showing a high-resolution approximation near the Talbot distance. Its applications are more paid attention in the non-paraxial propagation of grating structure. It can be used to observe defects in periodic or pseudo-periodic structures with complete paraxial approximation. Surface plasmons are electromagnetic waves propagating in the metal–dielectric interface^3^. These effects are mediated by plasmons and can be used in a wide range of applications, generating spatial frequency filters^4^, diffraction wave^5^, waveguide devices^6^, electron microscope imaging^7^ and diffraction X-ray^2^ has been investigated. Calculating diffraction patterns of the optical near field requires diffraction theory, which results in a complex integral and need numerical investigation. For this reason, attention is focused on some structures based on gratings beyond the paraxial limit (non-paraxial), one of the properties of Talbot effect is the formation of images in regular intervals and in a periodic structure despite the structural defects. However, the images are limited to the parabolic shape of the structure and the Talbot effect is restricted to the paraxial approximation. Talbot effect in the paraxial approximation is in the wavelength ranges far smaller than the Talbot distance; that’s why the Talbot images is simpler in this approximation. The non-paraxial approximation of Talbot effect in only with periodicity of the structure in one direction. In other words, paraxial approximation holds under ${a}_{0}/\lambda >>1$(a_0_ is grating period, λ stands for wavelength)^8^. There are challenges in constructing periodic patterns that can satisfy large-scale (${a}_{0}\sim \lambda$) design. Since the Talbot effect is an optical phenomenon with wave nature, it is expected that its effect can be observed in waves with surface plasmon. Surface plasmons are electromagnetic waves under diffraction that propagate along the joint surface between metal and dielectric and attract a lot of attention in optical manipulation. The wave nature of the surface plasmons motivated the scientists to investigate the Talbot plasmonic effect^2^.

## Plasmonic Talbot effect in meta-material

The Plasmonic Talbot effect is a phenomenon observed in metasurfaces or metamaterials that exhibit plasmonic behavior. It is named after the Talbot effect, which describes the self-imaging phenomenon of periodic structures. When a periodic object is at an angle higher than the diffraction limit, Talbot images can be observed when paraxial approximation is applied and the periodicity is considered greater than the diffraction limit; in other words, when the incident wavelength is larger than the periodicity the so-called super Talbot effect is created and it cannot be obtained from the paraxial approximation. This limitation is due to the instability of the wave propagation in the microwave range. The use of nanomaterials in the anisotropic media enables the observation of Talbot effect in the far IR range. This feature is due to the amplification of waves by polariton excitation of surface plasmons, and the vanishing waves are transferred to far-propagating waves. The simplest wave phenomenon is a material with far diffraction. However, the diffraction angle is significantly wider than the molecular collision, posing a great challenge for the development of far fields for objects that consist of several hundred thousand atoms. In this context, Zhao et al. investigated the effect of Talbot surface plasmons in a waveguide by placing an unknown metamaterial behind the wave medium at a wavelength of 160 nm with a periodicity of 2 µm without paraxial approximation. For the cases with period in the range of 50–100 nm, Talbot effect is not observed at the resonance wavelength of 630 nm due to the field decay. Therefore, Talbot effect is not observed in the cases where the period of the structure is negligible compared to the wavelength. Also, the measured transverse direction is approximately 160 nm, which is much smaller than 240 nm in air waves. It enables the formation of Talbot images in high frequencies in the range if far IR. No diffraction limitation exists in the super-Talbot effect for volumetric plasmons. Such that resolution below the deep wave of 0.08 incident wavelength can be achieved^10^. Since the propagation of the light wave does not depend on the light source, and it relies on the dielectric constant of the anisotropic material, Talbot image cannot be obtained. In further studies, the use of optical hyper-lenses made from a hollow cylinder of a hyperbolically dispersive metal/dielectric with multilayer metamaterial can process magnified optical imaging for a far-field sub-wavelength structure. Optical hyper-lenses depend on the type of material and the propagation frequency. The propagation frequency can be described in a small range of hyper-lenses^11^. When the light frequency is not equal to the plasma frequency, the emitted radiation is propagated in the sub-wave range with hyperbolic scattering in such condition. In this case, the image can be seen in a periodic object. Liang et al. The magnified Talbot image was investigated under diffraction where the dielectric constant changes relative to the changes in the anisotropic medium. Their proposed structure, from bottom to top, is made of chrome, dielectric metal and air with inner and outer radii of 500 and 1000 nm respectively, a thickness of 5 nm. In the case with incident wavelength of 441 nm, the inner grating is made of chrome. Radius is an effective parameter in Talbot revival, and Talbot revival time is reduced by increasing the image magnification. When the dielectric constant is constant and the permittivity of the metal is changed to a negative value of 1.5. The Talbot effect in waveguide structures can provide a homogeneous medium for the development of metamaterials^11^.

## Plasmonic Talbot Effect in Metal Nanostructures

**Figure 2:**
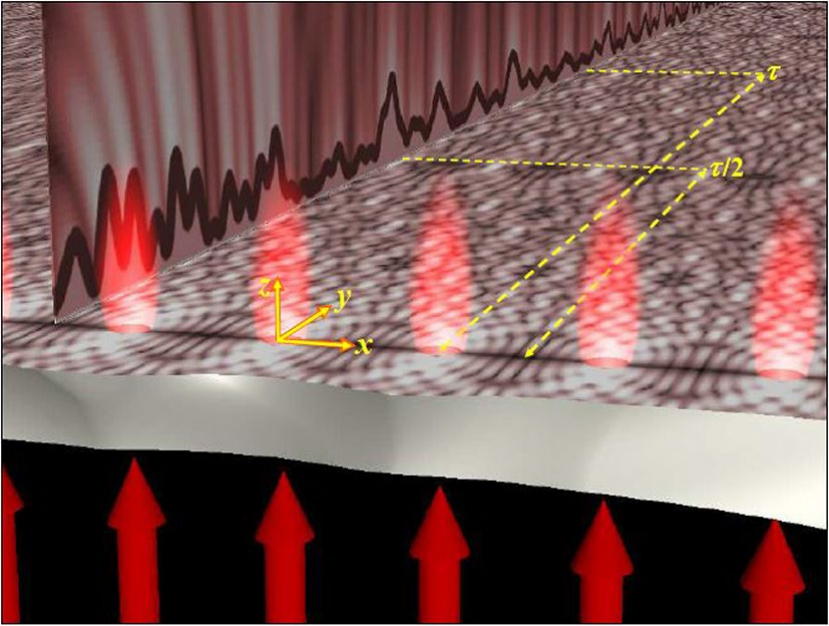
A schematic of plasmonic Talbot effect formation on the surface of a metal array^16^.

**Figure 3:**
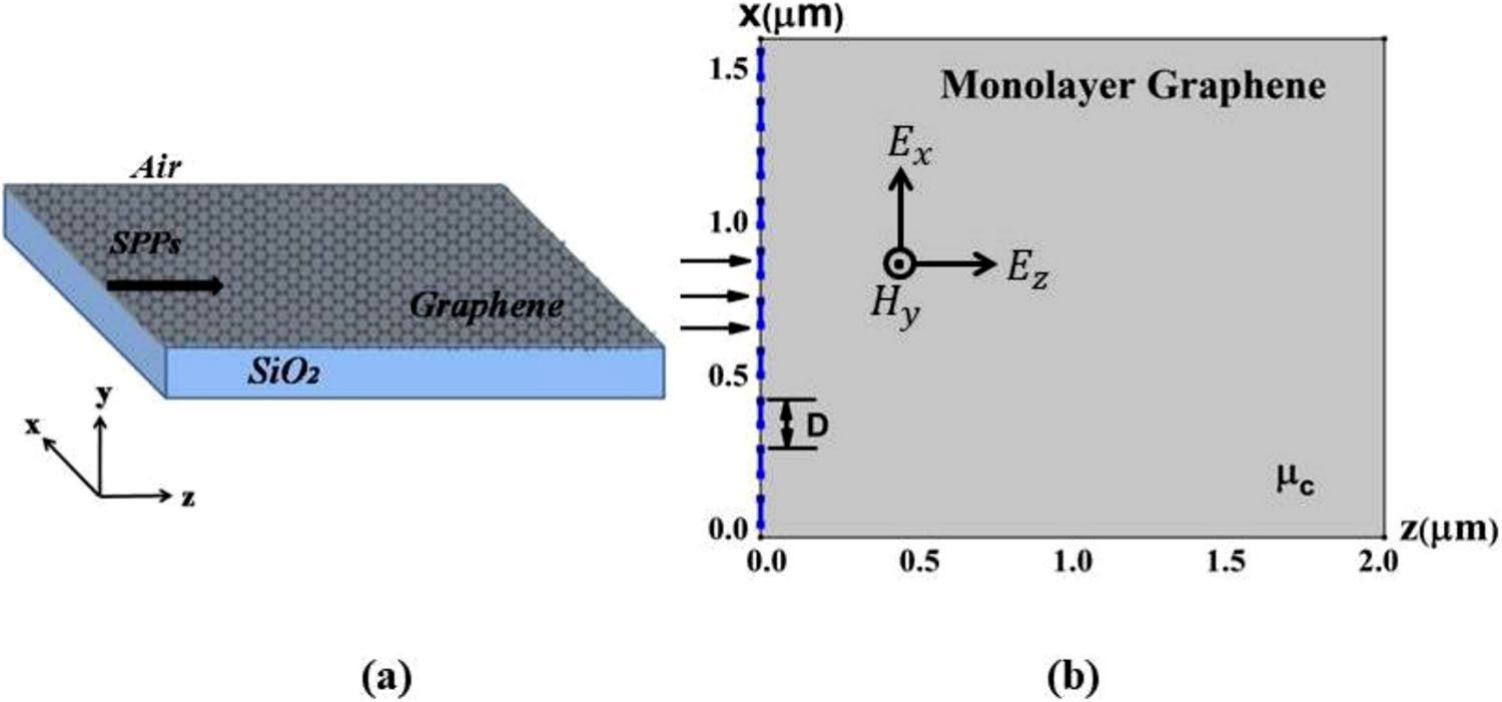
(**a**) structure when the graphene layer is surrounded by air dielectric on Silicon dioxide substrate, (**b**) a schematic of the graphene layer under the plasmonic Talbot effect^15^.

In recent years, the attention of researchers has been drawn to surface plasmon polariton and its wide applications in the near-field imaging. Electron oscillations at the metal–dielectric interface are accompanied by variations in the dielectric constant. When the light beam propagates on the metal surface, surface plasmon polaritons can realize the Talbot effect^12^. The studies show the surface plasmons can realize the Talbot effect. First observations of the Plasmonic Talbot effect were made by Denis et al. in 2007, when a planar wave of light collides a 1D periodic array of nano-tubes from the back of a metal layer. As shown in Fig. 2, a part of the light is transferred to the plasmons at the output of the substrate. Yu et al. investigated the Talbot effect in an array of metal nanostructures. They investigated three metal nanostructures including nanotube, nano-wave, and nanodots of gold metal on a pyrex substrate with a period of 500 nm (250 nm for nanotube) in air and water media with refractive indices of 1 and 1.33, respectively.” should be “Yu et al.^13^ investigated the Talbot effect in an array of metal nanostructures. They investigated three metal nanostructures including nanotube, nano-wave, and nanodots of gold metal on a pyrex substrate with a period of 500 nm (250 nm for nanotube) in air and water media with refractive indices of 1 and 1.33, respectively. The scientists found that the Talbot distance can be reduced to subwavelength. In this context, Wang et al. proposed periodic subwavelength arrays of metal waveguides made of silver layer and air gap, where the widths of silver substrate and air gap are one and two-dimensional, respectively, creating a planar wave of the magnetic field polarization perpendicular to the wave plane. A periodic distribution of the left end of the metal waveguide hits the subwave and excites the plasmonic field. However, a serious challenge in metal waveguides is the high attenuation caused by metal losses. The propagation lengths are short and depend on the wavelength and geometrical parameters of the waveguide, which vary in the range of tens to hundreds of microns. In general, low-loss plasmonic waveguides lead to wider fields in the surrounding medium, and vice versa. This problem is still an open issue for improvements for practical plasmonic waveguides and devices, therefore the Talbot effect is limited in the use of metal waveguide arrays. One of the challenges in the use of metamaterials in lenses is to achieve high image resolution at small dimensions. Recently, the Talbot effect at the surface of metallic nanostructures has been noticed. Metal nanoarrays produce Talbot revival in the far-IR range, which is due to the interference effect upon coupling the surface polaritons with the array of metallic nanostructures. Researchers have explored the focusing effect of gallium-doped zinc oxide nanoarrays functionalized by the coherent Talbot effect. while a silica membrane with a thickness of 100 nm has been used to increase transmission at a wavelength of 1550 nm^14^. The results showed that this material is a suitable alternative to plasmonic metal nanoparticles with lower losses. The scientists focused most of their attention on the graphene material. When the inter-graphene surface plasmon polaritons have a weak interaction, the plasmonic Talbot effect can be shown in single-layer graphene sheet arrays. This material has a two-dimensional structure which supports surface plasmon in the infrared and terahertz wavelength ranges. In addition to low inherent loss and high field confinement, it enables adjusting the chemical potential with the impurity content of the structure. Thus, the surface conductivity of graphene can be adjusted by controlling the chemical potential. Surface plasmon polaritons are excited and propagated by altering the surface conductivity of graphene. In other words, graphene-supported surface plasmon polaritons have lower loss and stronger field confinement compared to noble metals. This provides the possibility of determining the Talbot distance in the sub-wave range for imaging purposes. Honix et al. designed a structure based on silicon dioxide and single layer of graphene in which the Talbot distance is a function of the chemical potential of graphene. (Fig. 2) also shows the calculation of the plasmonic effect of the structure. The incidence angle is divided into n equal parts, and plane waves of unit amplitude in transverse magnetic mode with phase difference hit the surface periodically. In this case, adjacent incident waves with the same phase difference describe the grating wave function. Interference of all incident waves in each section increases light intensity. The grating period consists of two adjacent parts. Thus, The Talbot image propagates in a periodic structure. Contrary to the conventional optical Talbot effect, the plasmonic Talbot effect pattern exhibits attenuation properties which finally disappear along the propagation direction due to the loss of graphene emission. The angle of incidence is shown in (Fig. 3a). The wavelength decreases by 11 nm in which the Talbot effect is not observable. (Fig. 3b) plots the variation of the plasmon dielectric constant vs. wavelength at a given chemical potential. Longer wavelengths experienced more attenuation. Thus, the effective wavelength linearly increases with the wavelength. When the effective wavelength is equal with the period, the Talbot effect gradually disappears and the diffraction effect becomes weaker. As a result, the higher order diffraction wave is reduced by the grating in the sub-wavelength range. At this point, the Talbot effect disappears even if the wavelength continues to increase. As shown in (Fig. 2), the effective wavelength is 168.2 nm^15^.

The effective refractive index of the graphene layer at incident wavelengths is calculating using MATLAB^10^.

Metal plasmonic nanostructures, including nanowires, nanotubes, and nanoparticles, provide the possibility of surrounding waveguide light and reducing the scale for high-measurement applications. Due to their periodic structure, metal nanostructures have a different behavior between the resonance of surface plasmons in a grooved array and local surface plasmons with anomalies of Bloch and Wood type, so factors such as periodicity, thickness, shape and size of metal nanostructures are some of the influential factors in the interaction of light with matter. Yu et al. investigated the Talbot effect in an array of metal nanostructures. They investigated three metal nanostructures including nanotube, nano-wave, and nanodots of gold metal on a pyrex substrate with a period of 500 nm (250 nm for nanotube) in air and water media with refractive indices of 1 and 1.33, respectively.

When the wavelength is longer than the periodicity of metal arrays in three metal nanostructures, the results show that in gold metal nanowaves at the wavelength of 248 nm, as the concentration of the water sample decreases, a better resolution of the Talbot effect can be obtained by resonance of the surface plasmons, while for gold metal nanotubes, the shorter the wavelength, the better the Talbot images would be Ref.^16^.

Talbot length depends on the grating period and wavelength as^16,15^

1 $$S=\frac{2{a}_{0}^{2}}{{\lambda }_{0}},$$

where a_0_ refers to the period of the diffraction grating and λ_0_ refers to the wavelength of the light incident on the grating.

Our approximation considered the graphene is defined as an ultra-thin layer $d\to 0$ (d refers to thickness) of material characterized by a surface conductivity tensor. Thus, surface conductivity of graphene is modeled with Eq. (2)^15^.

2 $$ \sigma_{2D} = \mathop {\lim }\limits_{d \to 0} d\sigma_{3D} $$

In this paper, graphene layer is placed in free space in the x–y plane, where surface conductivity is calculated by Eq. (3)^15^.

3 $$ \sigma (\omega ,{\rm T},\Gamma ,\mu_{C} (E)) = \hat{x}\hat{x}\sigma_{xx} + \hat{y}\hat{y}\sigma_{yy} $$

where ω refers radian frequency, Γ refers scattering rate representing the loss mechanism, E refers to external voltage, and µc and T are chemical potential and temperature, respectively. Here, we consider the effect of the magnetic field to be zero. So, the surface conductivity of graphene is extracted from Kubo formula in a complex term consisting of interband and intraband. These terms (interband and intraband) are expressed in Eq. (4).

4 $$ \begin{gathered} \sigma_{{{\text{int}} ra}} = - j\frac{{e^{2} k_{B} T}}{{\pi \hbar^{2} \left( {\omega - j\tau^{ - 1} } \right)}}\left[ {\frac{{\mu_{c} }}{{k_{B} T}} + 2\ln \left( {e^{{{\raise0.7ex\hbox{${ - \mu_{c} }$} \!\mathord{\left/ {\vphantom {{ - \mu_{c} } {k_{B} T}}}\right.\kern-0pt} \!\lower0.7ex\hbox{${k_{B} T}$}}}} + 1} \right)} \right] \hfill \\ \sigma_{{{\text{int}} er}} = - j\frac{{e^{2} }}{4\pi \hbar }\ln \left( {\frac{{2\left| {\mu_{c} } \right| - (\omega - j\tau^{ - 1} )\hbar }}{{2\left| {\mu_{c} } \right| + (\omega - j\tau^{ - 1} )\hbar }}} \right) \hfill \\ \end{gathered} $$

In the present paper, e refers to the charge of the electron, k_B_ = 1.38 × 10^–23^ j/k refers to the Boltzmann constant, Γ = τ^−1^ refers to the dispersion rate, ħ refers to the reduced Planck constant, and µc refers to the chemical potential.

**Figure 4:**
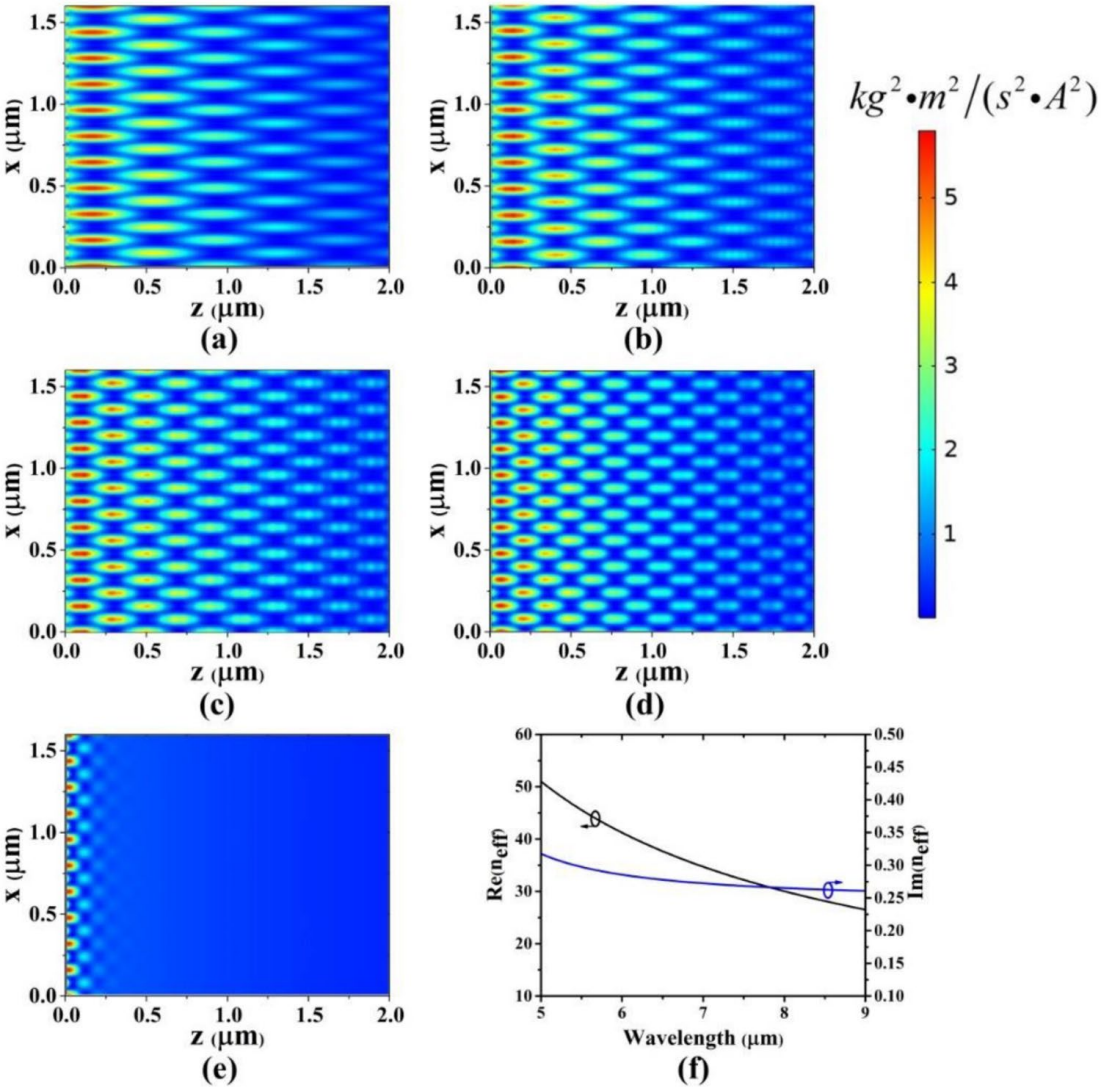
Plasmonic field intensity distribution of the graphene layer at a 7 um wavelength with period of (**a**) 160 nm. (**b**) λ_0_ = 8 um, (**c**) λ0 = 9 um, (**d**) λ0 = 10 um, (**e**) λ0 = 11 um, (**f**) imaginary part (described as Im (neff) = Im (β/k0))^15^.

Next, the scientists investigated the polariton effect of surface plasmons in Talbot effect revival in near fields. The rapid attenuation of polaritons reduces the propagation length. However, the Talbot distance in the plasmonic regime, in the paraxial Rayleigh approximation, was not simultaneous. In this context, Baville et al. designed the tunneling nature of surface plasmon polaritons in an infinite periodic grooved array on the silver substrate to observe the Talbot effect for investigating the effect of the geometrical parameters (e.g. periodicity, groove width, and groove thickness) in the near infrared range. When approximation is non-paraxial and the gap size of the silver metal array is smaller than the penetration depth of the surface polaritons, interference is formed behind the metal array and it is possible to create periodic images at certain intervals. The main factor of Talbot revival in the structure is due to the interference created behind the metal array, which has enabled the penetration of polaritons. In this structure, excitation of polaritons in the magnetic mode and the light comes to the surface from top. The presence of grooves can provide the required wave vector to excite the surface plasmon polaritons depending on the geometrical parameter. To better understand the effect of the geometrical parameters, the results have been analyzed. The field intensity changes for different gaps are shown in Fig. 4a–f. Talbot distance decreases rapidly as the altitude increases.

**Figure 5:**
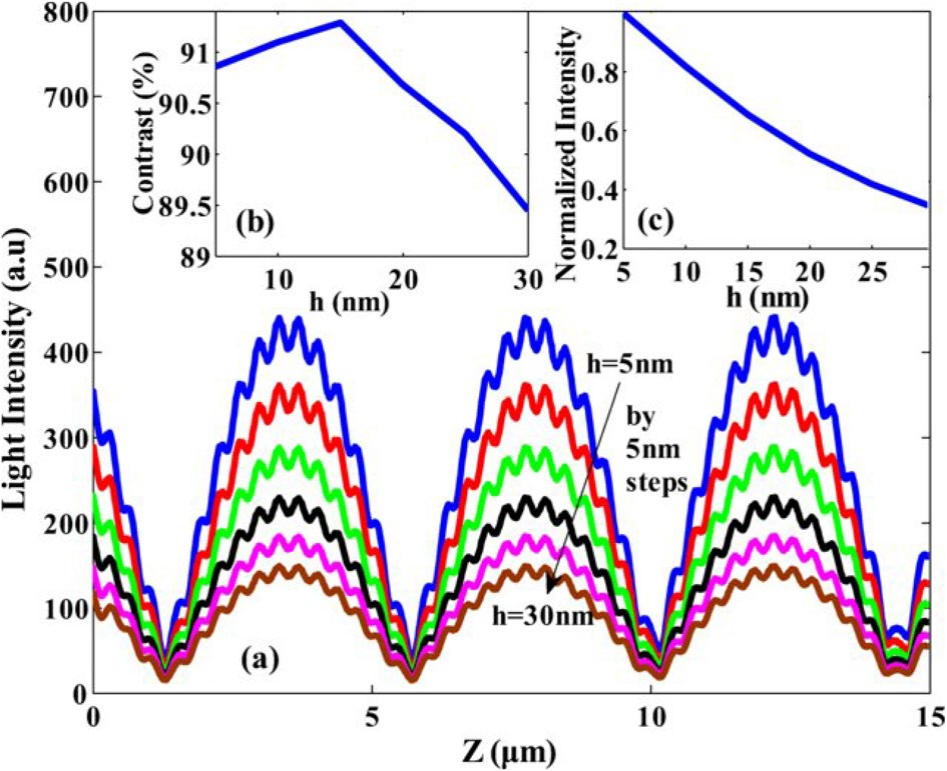
(**a**) light intensity changes vs. gap depth (changes have occurred at heights of 5 to 30 nm). (**b**) contrast changes (**c**) light intensity vs. thickness^18^.

**Figure 6:**
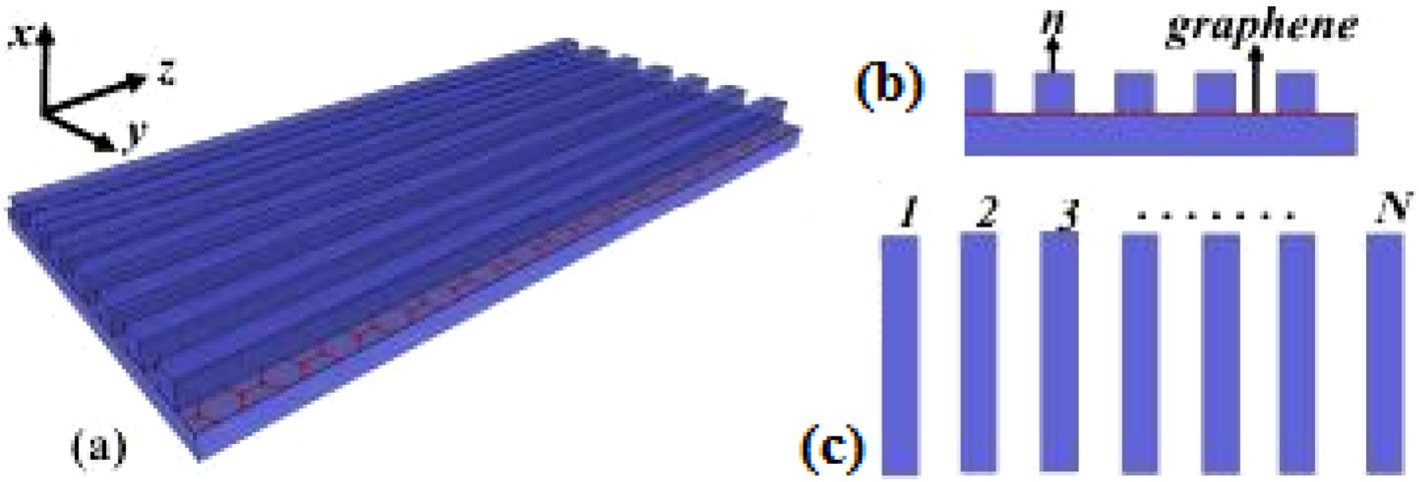
(**a**) Schematic of graphene structure, (**b**) a view of the graphene array cross section, (**c**) top view of the structure.

Next, the effect of light intensity on gap depth is inspected as shown in Fig. 5. Also, the maximum contrast changes are when the gap is equal to 15 nm, which is shown in (Fig. 5a), while the revival changes determined by R^2^ show gradual changes regarding the contrast of the image, which is shown in (Fig. 5b). So, when the gap is between 15 and 20 nm, Talbot revival is better formed. Also, light intensity versus thickness is calculated in Fig. 5c. The period is fixed, any change in the period value can change the thickness of the grooves at the wavelength of 1220 nm and 632.8 nm, respectively. These changes are proportional to the periodicity of 0.2 to 0.7 in the direction perpendicular to the structure^15^. It is created under the self-imaging effect in the paraxial approximation and in periodic structures, while in the metal nanostructures that are defined beyond the paraxial limit, the self-imaging effect does not create the same periodicity compared to the image pattern in comparison with the incident wavelength, so Therefore, images in different non-paraxial optical frequencies do not appear in the Talbot distance. Furthermore, Kim et al. investigated the Talbot effect of gold nanoparticles in the paraxial approximation. Using differential contrast based on interference, they observed the effect of non-paraxial approximation in gold nanoparticles. Gold nanoparticles with a period of 600 nm and a diameter of 120 nm were placed in water with a refractive index of 1.33., then obtained the interference pattern of the polarized light beam from the laser with the periodic structure of gold nanoparticles in the state of interaction at the excitation wavelength of 600 nm, they obtained the dark and light areas caused by light beam and nanoparticles interaction at the height of 50 nm, leading to the resonance peak of local surface plasmons^17^. The effect of surface plasmons in a structure based on grooved silver metal nanoparticles which their gap is smaller than the penetration depth of surface plasmons is separated from surface plasmons based on the non-paraxial approximation. Plasmons penetrate deep into the metal nanostructure and cause interference. In this case, the images are formed periodically at the Talbot distance. The results show that increasing the intensity of the irradiated light does not change with increasing the Talbot distance; in other words, the groove depth does not affect the Talbot distance, but it depends on the irradiated light. Figure 6 shows the light intensity changes vs. Talbot distance. The light intensity is decreased compared to the changes of the revival parameter.

Thus, when gap is small and the height ranges between 15 to 20 nm, higher-resolution images are obtained. Investigating the increase of silver thickness in the range of 50 to 300 nm does not affect the Talbot contour significantly. Therefore, the substrate thickness is fixed at 150 nm. Figure 6 shows that contrast at 15 nm gives the best performance with a maximum height of 5 nm. Li et al. investigated the Talbot effect in graphene dielectric waveguide arrays to change the Fermi energy of graphene in the terahertz range. The changes in the Fermi energy of graphene depend on the Talbot distance, so that increasing the number of graphene dielectric waveguide arrays enhances the Talbot distance and at a wavelength of 10 µm for discrete Talbot imaging, lower absorption loss is created for resonance of surface plasmons in the propagation mode with low atmospheric losses. In this structure, the dielectric waveguide is placed on graphene layers with width and height. The relative permittivity of graphene in this structure is chosen 3.92 with an air layer coating with a dielectric constant of one, where its 2D structure is shown in (Fig. 6a–c). The results show that the Fermi energy changes with the field components {1, 0, 1, 0, 1, 0, 1} and {1, 0, 0, 1, 0, 0, 1}, reducing the Talbot distance^18–21^.

**Figure 7:**
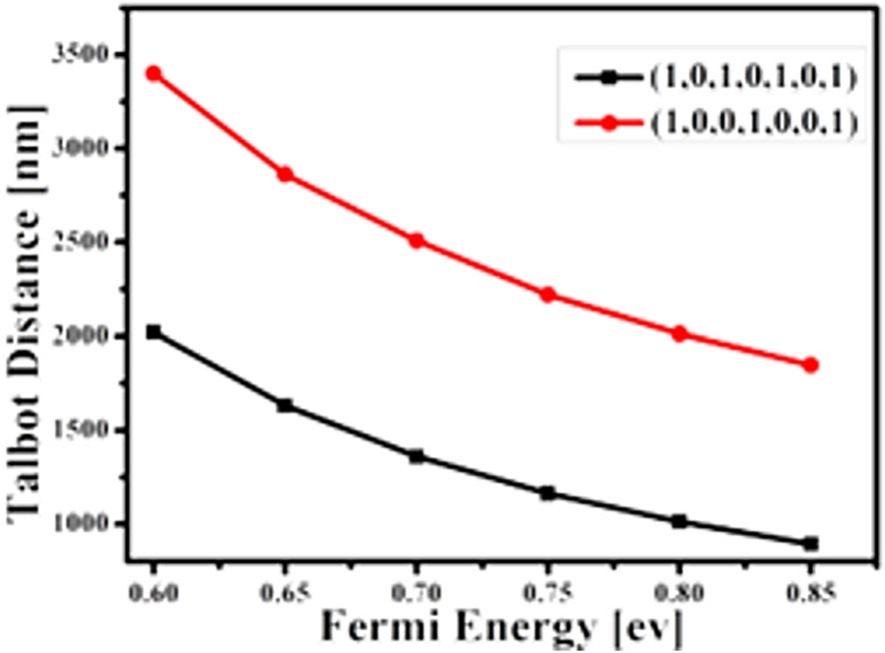
Talbot displacement vs. Fermi energy^19^.

Talbot displacement vs. Fermi energy is calculated in Fig. 7. As shown, as Fermi energy increases with random periods, the Talbot distances decreases.

As shown, as Fermi energy increases with random periods, the Talbot distance decreases.

## Application of Talbot effect in plasmonic structures

Talbot displacement is one of the imaging techniques in lithography, which is used to create periodic submicron features in areas of 200 mm diameter wafers for plasmonic and metamaterial applications. The Talbot effect in lithography is based on incident interference for periodic masks that their periodicity is smaller than the Talbot effect such that by reconfiguring the mask, uniformity and contrast based on Talbot effect can be controlled. Periodic nano-diaphragms in thin metallic films have shown a range of interesting properties in the presence of an electromagnetic wave, such as the extraordinary optical transmission of electron beam lithography. These effects are mediated by plasmons and can be used in a wide range of applications, such as color filtering and chemical sensing. Lithography techniques are limited to electronic and ionic beams, which cover micron dimensions. However, the main drawback of focused ion beam lithography is the slow patterning speed, limiting access to operating fields with dimensions of 100 × 100. For fabrication at dimensions below 100 nm, low-ion beam currents (~ 100 pA) are often used, which limit the possibility of large-scale fabrication. The electron beam method also requires patterning in large areas and creating a constant field pattern (~ 33 × 26) for limited illumination, which Large-scale manufacturing is costly and suffers from high complexity. Balavera et al. presented a straightforward method for making large-scale plasmonic structures due to light transmission in the Talbot displacement lithography method. This technique is effective in designing square, rectangular, and hexagonal nanoarrays. Such that it can be adjusted by changing the frequency of the exposure mask. Also, the transmission properties are significantly improved by increasing the thickness of the metal layers. Hong et al. studied the effect of laser reflection on photonic crystals. Where higher-order nonlinear images are subjected to the laser beam, a magnified image of surface plasmons on a silicon surface shows Talbot transient reflection images caused by electrons floating on the plasma surface^22–25^. Some of the researches conducted in the Talbot effect plasmonic are given in Table 1.

**Table 1:** Some of the studies conducted on the plasmonic Talbot effect.

References	Structure	Application
^13^	arrays of metallic nanoapertures	Roughness detection in alternating structures
^26^	monolayer graphene sheet arrays	increasing the chemical potential of graphene
^27^	waveguide metallic nanostructures	The exact positions of the field distribution
^28^	nanowire arrays	Reduce ohmi losses
^29^	grating	high-resolution structures with high throughput
^30^	wire waveguides	create periodic microparticle traps to microturbine systems
^22^	nanostructured metal electrodes / ITO free thin film	electrodes and the epoxy encapsulation
^25^	Cytop polymer grating layer/air	biomaterials detection Ether, Ethyleneglycol, Chlorobenzene and Quinoline
^31^	grating	near-field diffraction region

All abovementioned references from Ref.^13^ to Ref.^31^ have several pros such as compact structure, high stability and sensitivity to environmental effects and their tunability.

Some practical Talbot effect applications:

## Arrays of metallic nanoapertures for Talbot effect

When light interacts with metallic nanoapertures, it undergoes various optical behaviors, such as diffraction, interference, and plasmonic effects. These phenomena are dictated by the size, shape, and arrangement of the nanoapertures. In a high-index medium, the Talbot effect is enhanced due to the increased confinement and manipulation of light within the nanoapertures. This enhances the diffraction and interference patterns generated by the periodic array, resulting in more pronounced self-imaging effects.

The exact behavior of the Talbot effect in such systems can be influenced by multiple factors, including the geometry of the nanoapertures, the wavelength and polarization of the incident light, and the refractive index of the surrounding medium. Additionally, plasmonic effects can interact with the Talbot effect, leading to further control of the transmitted or scattered light.

**Figure 8:**
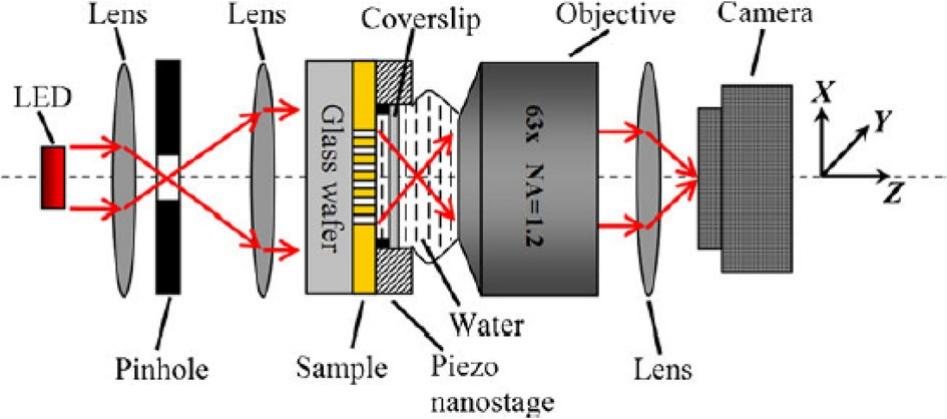
Experimental setup of Talbot effect in arrays of metallic nanoapertures^13^.

Understanding and harnessing the Talbot effect in metallic nanoaperture arrays in high-index media have important implications for various applications. These include optical imaging, lithography, beam shaping, and sensing, where the self-imaging phenomenon can be utilized for subwavelength imaging, nanostructuring, and manipulating light at the nanoscale. The experimental setup is shown in Fig. 8.

## Plasmonics biosensors based on the Talbot effect

**Figure 9:**
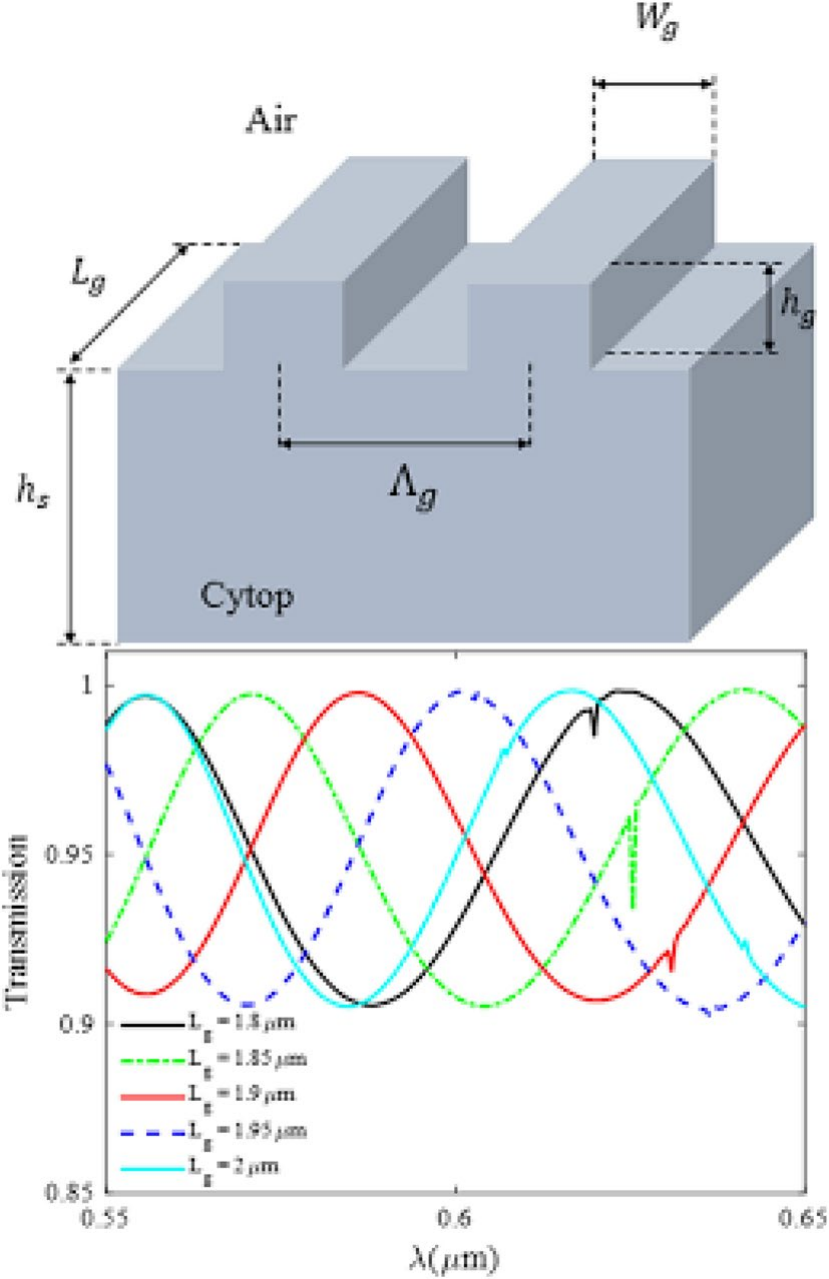
Plasmonics Talbot biosensors.

Surface plasmon resonance (SPR) biosensors based on the Talbot effect are advanced tools used in bioanalytical applications (see Fig. 9). These biosensors leverage the principles of SPR and the Talbot effect to enable highly sensitive and label-free detection of biomolecular interactions. The Talbot effect, discovered by Henry Fox Talbot in the nineteenth century, is an optical phenomenon that occurs when coherent light passes through a periodic structure, such as a diffraction grating. The light waves interfere with each other, creating a self-imaging pattern at regular distances known as Talbot planes. In an SPR biosensor based on the Talbot effect, a diffraction grating is integrated into the sensor chip. This grating is designed to create an array of periodically spaced nanoslits or nanogrooves. When light is incident on this grating, it generates a Talbot self-imaging pattern. To measure biomolecular interactions, a thin metal film (typically gold or silver) is deposited on the chip surface. When light at a specific wavelength is coupled with the metal film, it generates surface plasmon waves at the metal–dielectric interface. The presence of biomolecules on the metal surface affects the local refractive index, altering the surface plasmon waves.

The Talbot effect comes into play by ensuring that the patterned grating maintains its periodicity regardless of the angle of incidence. This allows for precise control of the incident light's angle and ensures consistent excitation of surface plasmons across the sensor chip. By monitoring the changes in the reflected or transmitted light intensity as biomolecules bind to or dissociate from the metal surface, the SPR biosensor based on the Talbot effect provides real-time information about the kinetics, affinity, and concentration of analytes in a sample. This technology has numerous applications in fields such as medical diagnostics, drug discovery, and environmental monitoring.

### Fractional nonparaxial accelerating Talbot effect

**Figure 10:**
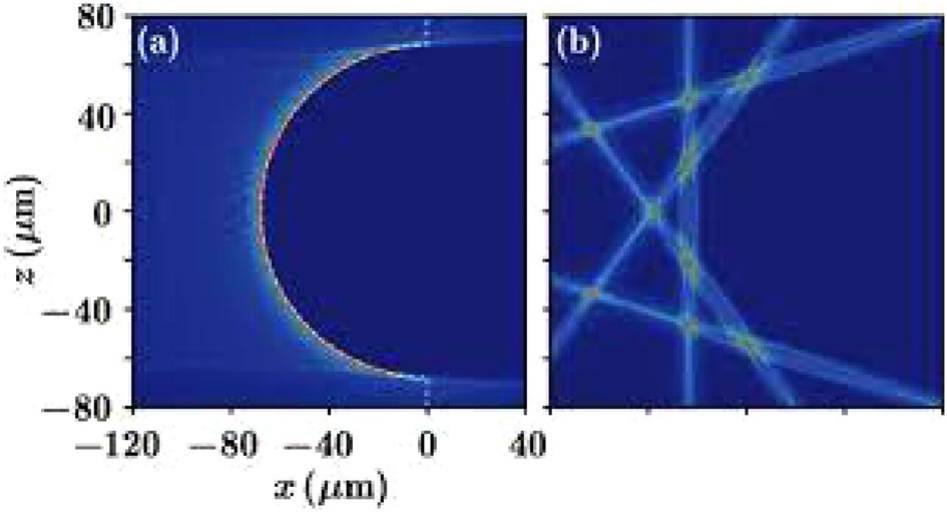
Fractional nonparaxial accelerating Talbot effect^29^.

The fractional nonparaxial accelerating Talbot effect is a phenomenon related to the diffraction of light waves. It refers to the fractional revival patterns of intensity distribution that occur at specific distances behind certain periodic structures, such as diffraction gratings. In the paraxial approximation, the Talbot effect describes the self-imaging phenomenon where the intensity profile of an incident wavefront reconstructs itself at integer multiples of a characteristic distance known as the Talbot length. However, in the nonparaxial regime, where the angles of incidence and diffraction are large, the conventional Talbot effect is no longer strictly applicable, and fractional Talbot revivals emerge (see Fig. 10).

The fractional nonparaxial accelerating Talbot effect occurs when the fractional order of the revival pattern deviates from the exact integer multiples. This deviation arises due to the nonparaxial nature of the incident beam, resulting in a modification of the characteristic distance and intensity distribution.

This effect has found applications in various areas, including beam shaping, optical trapping, and microparticle manipulation. By understanding and exploiting the fractional nonparaxial accelerating Talbot effect, researchers can design novel optical systems with unique properties and functionalities.

For example, the Talbot effect can be employed in the fabrication of plasmonic gratings. By carefully designing the diffraction grating, the periodic pattern formed by the Talbot effect allows for precise control over the distribution and intensity of plasmonic modes. This enables the manipulation of light at subwavelength scales, which is crucial for applications such as nanophotonic circuits, sensing, and imaging.

Additionally, the Talbot effect can be used in the characterization and measurement of plasmonic devices. The self-imaging property of the Talbot effect provides a method to analyze the periodicity and quality of plasmonic structures^32–45^. This is essential for assessing the performance and optimization of plasmonic devices in various applications^46–57^. In this realm recently several invaluable practical were proposed^58–64^.

## Perspectives

Plasmonic Talbot effect occurs due to the high capability of plasmons in the range of electromagnetic field, however, observing this effect has always been challenging. One of the challenges is plasmon propagation over long distances. Further studies revealed that metamaterials as materials with negative refractive index enables the propagation of plasmons to extend the Talbot effect in far fields. The reason for the strengthening these waves is the surface plasmon polaritons excitation, transferring vanishing waves to far-propagating waves. This feature is due to the super Talbot effect in smaller periods of the wavelength. Despite the presence of the Talbot effect in the planar anisotropic metamaterial, the magnified image cannot be obtained as the propagation characteristic of the light wave does not depend on the light source and rather relies on the anisotropic permittivity of the material. In such cases, the use of cylindrical hyper-lenses can offer magnified optical imaging for a sub-wavelength structure in the far field. Another challenge of the Talbot effect in metal structures is due to ohmic losses for the propagation of plasmons over long distances. Researchers have proposed using some metal nanoparticles such as doped oxide functionalized with gallium and graphene as a plasmonic alternative material. Graphene is used as a functional material in many structures. Talbot plasmonic effect can be shown in single-layer graphene sheet arrays. It supports surface plasmon in the infrared and terahertz wavelength range. In further studies, the researchers investigated the effect of geometrical parameters on the Talbot effect, the size of the groove, the period and the thickness of the gap on the light intensity in the Talbot effect using surface plasmons. The necessary wave vector for exciting the surface plasmon polariton can be created by the grooves depending on the geometric parameter and the wavelength of the incident light. So that in metal arrays, the thickness of the layer has a significant effect on the reflected light, and the light intensity has no effect on increasing the Talbot distance, however, the periodicity is effective in the Talbot distance proportional to the thickness changes, and can be effective in increasing the propagation of surface plasmons. Also, the effect of the gap size in metal nanoarrays is effective in the tunneling rate of surface plasmons proportional to the Talbot distance. The studies conducted in the field of Talbot effect in recent years have progressed to the use of metamaterials for the propagation of polaritons in far fields. To maintain the clarity and magnification of the image while propagation in wider wavelengths, it is expected that further developments of nanomaterials based on their non-linear properties will be proposed to increase Talbot imaging.

## Conclusion

Here, graphene nanostructures with theoretical models is applied for plasmoincs Talbot effect. By application of a single graphene grating layer, the Talbot effect for the plasmonics applications in the IR range is theoretically studied and the respective Talbot effect for the novel advanced plasmonics structures are numerically investigated for the first time. It was shown that the metamaterial structures with periodic grating configuration represents a complex three-dimensional lattice of beamlet-like graphene plasmonics devices. The calculated results agree well with the experimental ones. The results obtained were used to create and optimize the structures considering diffraction limit for a wide range of application areas. Effective focusing of plasmonic waves with exact focal spots and a subwavelength full width at half maximum can be obtained by using periodic graphene grating.

## Data Availability

All data included in this paper are available upon request by contact with the contact corresponding author.
